# Momentary predictors of a broad range of food parenting practices within a population-based sample of parents of preschool-aged children

**DOI:** 10.3389/fpubh.2022.944734

**Published:** 2023-01-16

**Authors:** Katie A. Loth, Ziyu Ji, Julian Wolfson, Jennifer Fisher, Jerica Berge, Dianne Neumark-Sztainer

**Affiliations:** ^1^Department of Family Medicine and Community Health, University of Minnesota Medical School, Minneapolis, MN, United States; ^2^Division of Biostatistics, School of Public Health, University of Minnesota, Minneapolis, MN, United States; ^3^Department of Social and Behavioral Sciences, College of Public Health, Temple University, Philadelphia, PA, United States; ^4^Division of Epidemiology and Community Health, School of Public Health, University of Minnesota, Minneapolis, MN, United States

**Keywords:** food parenting practices, preschool-aged child, ecological momentary assessment (EMA), stress, mood, child behavior

## Abstract

**Introduction:**

The current study sought to understand the influence of momentary factors within the home and family environment, including parent stress, parent and child mood and child behaviors, on parents' use of a broad range of food parenting practices later that same day.

**Methods:**

Ecological Momentary Assessment (EMA) was used to evaluate parents' use of coercive, indulgent, structured and autonomy support food parenting practices, as well as numerous potentially salient momentary predictors, including parental stress, parent and child mood, and child behavior. Data were collected from 109 parents of preschool aged children multiple times per day over the course of a ten-day data collection period, allowing for temporal sequencing of momentary predictors and use of food parenting practices.

**Results:**

With some notable exceptions, study findings align with study hypotheses in that parent stress, parent and child low mood, and child negative behaviors early in the day were found to be associated with the use of less supportive food parenting practices later that same day. For example, greater parent negative mood earlier in the day was associated with a decrease in use of feeding practices from within the structure domain later on that same day (−2.5%, *p* < 0.01), whereas greater parent positive mood earlier in the day was associated with an increase in use of structure later on that same day (+3.7%, *p* < 0.01). Greater parent stress earlier in the day was associated with an increase in the use of coercive control (+3.2%, *p* < 0.01) and indulgent (+3.0%, *p* < 0.01) practices later that same day; surprisingly, a similar increase in stress earlier in the day was also found to be associated with an increase in the use of autonomy support (5.6%, *p* < 0.01) feeding practices later on that same day.

**Discussion:**

Developing an understanding of the types of momentary factors that influence a parent's use of particular food parenting practices across multiple contexts is a crucial next step toward developing effective interventions aimed at teaching parents to use food parenting practices that are supportive of healthful child dietary intake and eating behaviors in a way that is responsive to shifting factors.

## Introduction

Healthful eating patterns and dietary intake during early childhood are important for growth and development and for the long-term prevention of health concerns (1). Children's eating behaviors and dietary intake are shaped significantly by their family and home food environment (2–5). Parents use a broad range of food parenting practices, or goal-oriented actions and behaviors to shape and socialize their children's eating behaviors and dietary intake (2, 6–9). For example, a parent might engage in food restriction (i.e., limiting the types or amounts of foods their child can eat) with the goal of helping them to avoid overconsumption of certain foods, or a parent might engage in pressure-to-eat feeding practices in response to challenges associated with feeding their child that struggles with pickiness. Food parenting practices encompass a range of behaviors that dictate what foods are made available and accessible to their child, as well as the nature and tone of interactions with children around food (2, 9). The current research study draws from a leading conceptual framework of food parenting, developed by Vaughn and colleagues, which describes three higher-level domains of practices: structure, such as food availability, accessibility, and limit setting; autonomy support, such as praise and reasoning; and coercive control, such as pressure-to-eat and overt food restriction (2, 9). Indulgence has been proposed to be either a sub-domain of structure (2), or a fourth unique high-level domain of potential importance (6); indulgent behaviors allow children greater freedom over what, when, and how much to eat. Current theory and research to date suggest that food parenting practices within the structure and autonomy support domain are “supportive” and those practices within the coercive control and indulgent domain are “unsupportive” of healthful dietary intake and eating behaviors in children (2–5). However, empirical evidence to support the impact of food parenting practices within the structure and autonomy support domains on child outcomes is much more limited than the evidence-base examining the short- and long term impacts of coercive control practices (2–5, 9). For example, many structure and autonomy support food parenting practices have received little [e.g., guided choices (2)] or no [e.g., food preparation (2)] attention in prospective studies, (2, 5) with other structure practices having a much deeper evidence base (e.g., food accessibility, availability, modeling) (4, 5).

There is a large body of literature that indicates that high levels of parental stress and parental depressed mood are associated with a parent's own unhealthy dietary intake and less healthful food preparation (10, 11). Less is known about how the relationship between parental stress and mood and parental use of specific food parenting practices (12). A small number of research studies published in the extant literature have revealed associations between maternal depressed mood and use of pressure-to eat feeding practices (13) and maternal stress and use of controlling food parenting practices (12), suggesting that these individual parent-level factors do influence parent's engagement in food parenting practices. However, although experts generally agree that food parenting practices are goal-directed behaviors sensitive to circumstance, previous studies have only typically assessed parents' “usual” use of food parenting practices *via* questionnaires, failing to account for potentially important variation in use of specific food parenting practices across time and contexts (2, 8). For example, a parent might report *via* survey their “usual” use of coercive feeding practices is low or infrequent, but on days when their stress level is particularly high or their child's eating behaviors are highly challenging, they might pressure children to eat particular foods or place greater restrictions on children's eating. Indeed, our recent research provides new preliminary evidence that food parenting practices show significant within- and between-day variation that is shaped by a wide range of momentary influences encountered in everyday family life (6, 14, 15). Specifically, in our prior qualitative research parents of preschoolers described a number of momentary factors that influenced their use of specific food parenting practices: parent mood or stress level, child mood, behavior or physical health, time constraints, lack of planning, and/or competing priorities (e.g., other children, job requirements, activities or special events) (6). Importantly, parents in this study described shifts from the use of structure- and autonomy support- feeding practices to more indulgent and controlling practices in the face of external challenges highlighting the need for more nuanced approaches to investigating potential sources of within- and across-day variation in food parenting practices. Findings from this study (6) support the premise that challenges experienced early on-, and throughout- the day contribute to a parent's differential use of specific food parenting practices at shared meals later in the day. While not directly examined within this qualitative study, it seems that external challenges leading to parents running out of time, patience, or energy might be contributing factors to the shift in approach described.

Recent evidence on temporal relationships between these momentary variables (e.g., stress, child behavior) and use of food parenting practices provides some support for this perspective (16, 17). Indeed, two recent publications by Berge and colleagues provided preliminary quantitative evidence of these momentary (i.e., within- or between-day) shifts in food parenting practices through the use of Ecological Momentary Assessment (EMA) (16, 17); EMA uses short surveys delivered to hand-held devices in real time throughout the day to capture dynamic changes in behaviors across time and context (18). Specifically, Berge and colleagues found that high levels of parental stress, as well as parental depressed mood, earlier in the day were found to predict greater use of coercive control feeding practices later the same day (16, 17). This preliminary work demonstrates that momentary influences experienced early in the day can shift parents' engagement to food parenting practices that are unsupportive and associated with higher risk of poor dietary intake over time. That said, little is known about how various momentary factors, including child-related factors (e.g., child mood, behavior) influence the within-day variability in parents' use of food parenting practices that fall within the structure-, autonomy support-, and indulgent- higher-order domains (15).

Thus, the current study seeks to build upon and extend this early work by seeking to understand how parent and child mood, parent stress, and child behaviors early in the day are associated with parent use of a broad range of specific food parenting practices, situated within four higher order domains (structure, autonomy support, indulgent, coercive control), later that same day. Furthermore, the current sample includes young children ages 2–5, whereas, Berge's studies included children ages 59 (16, 17). Thus, the current study advances the science of examining momentary predictors of food parenting with preschool children. Based on findings from our previous qualitative work with parents of young children (6), we hypothesized that greater negative mood (parent and child), high stress, and negative child behavior early in the day would be associated with greater use of coercive control and indulgent food parenting practices later on that same day, whereas greater positive mood (parent and children), lower stress, and positive child behavior early in the day would be associated with greater use of structure and autonomy support food parenting practices later on that same day. To our knowledge this is the first study to examine the impact of multiple momentary influences (parent- and child-level) on the use of such a broad range of food parenting practices, including practices from across the four higher-order domains most commonly discussed in current conceptual models of food parenting practices (structure, autonomy support, coercive control, indulgence) with preschool children, within a sample of preschool-parent dyads. Findings from our research studies to date underscore the importance of considering food parenting practices as context specific and responsive to changes in the home environment, including stress and mood. Developing an understanding of the types of momentary factors that influence a parent's use of particular food parenting practices across multiple contexts is a crucial next step toward the development of just-in-time adaptive interventions, or interventions that aim to deliver intervention content to participants' mobile devises in response to real-time assessments of context, behavior and circumstance. Long term, findings the from the current study will inform the design of just-in-time adaptive interventions developed with the goal of improving children's dietary intake and eating patterns and consequently reducing the morbidity and mortality associated with chronic disease across the life span.

## Materials and methods

### Study design and population

Data for the present study are from Kids EAT!, mixed-methods observational study designed to deepen our understanding of parents' experiences feeding their preschool-aged child and the factors that influence their decisions about feeding (14). Kids EAT! study participants (*n* = 116) completed traditional questionnaires about demographics, family routines and functioning, and child feeding and eating behaviors *via* online surveys, followed by 10 days of ecological momentary assessment (EMA) completed *via* cell phone during the fall of 2019. The current study only uses data from the EMA data collection protocol.

### Study population, recruitment, and participant demographics

Kids EAT! (14) is an ancillary study to EAT 2010–2018 (Eating and Activity among Teens) (19) a large, population-based cohort study on eating and weight-related health. Survey data collected from 1,491 young adults (Mean age 22.2) as a part of EAT 2018 were utilized to identify potential Kids EAT! participants that met the inclusion criteria; young adults who indicated on the EAT 2018 survey that they had at least one child aged 2–5 years who lived with them at least 50% of the time were invited by email to participate in the Kids EAT! study. Participants in the original EAT 2010–2018 cohort lived in the Minneapolis—Saint Paul metropolitan area during their initial participation in 2010; eligible participants were invited to participate in Kids EAT! regardless of their current geographic location at the time of data collection for this study. Kids EAT! recruitment e-mails indicated that the study goal was to learn more about parents' experiences feeding their pre-school aged child and provided some information about study data collection procedures. The University of Minnesota's Institutional Review Board Human Subjects Committee approved all protocols used for the Kids EAT! study.

Table 1 provides demographic information on the sample. The participating parents (*n* = 109) had a mean age of 26.4 at the time of survey completion. Just over half of participants (56%) reported education beyond high school. Approximately 21.1% of the sample reported household incomes below the 2020 Federal Poverty line for household sizes of 2 or more individuals ($17,420) (20).

**Table 1:** Study demographic characteristics (*n* = 109).

		**n**
Parent gender	Female	91 (83.5)
	Male	18 (16.5)
Parent race/ethnicity	Black	35 (32.1)
	Hispanic	26 (23.9)
	Asian	19 (17.4)
	White	16 (14.7)
	More than one race/other	9 (8.3)
	Native American	4 (3.7)
Parent education	Partial high school or less	11 (10.1)
	High school graduate or GED	37 (33.9)
	Partial college or specialized training	39 (35.8)
	College graduate	19 (17.4)
	Graduate degree	3 (2.8)
Spouse education	Partial high school or less	10 (9.2)
	High school graduate or GED	31 (28.4)
	Partial college or specialized training	22 (20.2)
	College graduate	9 (8.3)
	Graduate degree	5 (4.6)
	No spouse/not applicable	32 (29.4)
Household income	$0–$9,999	16 (14.7)
	$10,000–$14,999	7 (6.4)
	$15,000–$24,999	20 (18.3)
	$25,000–$34,999	21 (19.3)
	$35,000–$49,999	16 (14.7)
	$50,000–$74,999	20 (18.3)
	$75,000–and above	9 (8.3)

### Procedures and data collection

Participants completed the Kids EAT! baseline survey online, using an individualized link included in the study recruitment e-mail. The survey included questions on a wide range of topics including demographics, family routines and functioning, and child feeding and eating behaviors. Next, parents were given detailed instructions for how to complete the EMA protocol. The 10-day EMA data collection period began the day following survey completion. Standardized EMA data collection protocols from prior studies (18, 21, 22) were used to guide the development of EMA-based *Real-Time Feeding Practices* survey (14) and sampling methods.

During the EMA data collection period, parents were asked to complete surveys in response to three types of EMA sampling methods: (1) signal-contingent, (2) event-contingent, and (3) end-of-day EMA surveys. Parents completed all EMA recordings using their own electronic device (i.e., cell phone, tablet) using a link provided to them *via* SMS text message. On average, each EMA recording took participants 2–3 min to complete.

Parents were sent four signal-contingent surveys per day. Signal-contingent surveys were spaced so they began after the parent woke up (information provided prior to starting EMA). The time between the parents' reported wake and sleep times was divided into five blocks to accommodate the semi-random scheduling of 4 signal-contingent surveys and the end-of-day survey, with at least 1 h separating each block (e.g., a block of time from 8 to 11 AM with the next block starting at noon), so that there would never be an overlap of surveys. Scheduling signal-contingent surveys around the parents sleep and wake time allowed surveys to be scheduled to accommodate different life situations (e.g., working an overnight shift), if needed. Parents were notified *via* SMS text message that a signal contingent survey was ready to be taken; they would click the link provided which would take them to a secure web-based survey. Signal contingent surveys measured parent stress, parent and child mood, and child behavior. Specific measures used in analysis for the current study are described in detail below.

Event-contingent surveys were self-initiated by parents whenever the child ate in the presence of the parent (i.e., both meals and snacks); importantly, parents did not need to be sitting and eating with the child to complete a recording, they were only required to be present to the degree that they felt they could respond to the questions specific to the eating occasion. It was important to have parents fill out the EMA response even when they were not eating with their child because parents often still engage in food parenting practices in this situation. To initiate an event-contingent survey parents clicked on a link provided to them *via* SMS message; this link remained the same throughout the EMA data collection period allowing parents to use the same link throughout the full study period to respond to all event-contingent recordings. Knowing that participants might forget to report a shared eating occasion, at the start of each signal-contingent survey they were asked about—and given the opportunity to report on—any shared eating occasions that they may have failed to report on. Event contingent recordings asked parents to report on details of the eating occasion that prompted the recording, including their use of specific food-related parenting practices. Specific measures used in analysis for the current study are described in detail below.

A link to complete the end-of-day survey was provided to parents *via* SMS text message in the hour prior to their reported typical sleep time. Data from end-of-day surveys were not used for the current analysis so they are not described in further detail.

All EMA surveys were completed in English; participants' English language fluency was determined during their initial enrollment in the EAT 2010–2018 study. Families were offered an incentive of a $150 gift card for participation in the Kids EAT! Study. Data collection was completed on all participants between October 2019 and February 2020.

### Measures

*Food parenting practices* were measured during EMA event-contingent surveys using the EMA-based Real-time Parent Feeding Practices survey tool (14). This tool was developed for the Kids EAT! Study, based on prior validated measures if available, to measure a broad range of food-related parenting practices within an EMA protocol. The survey includes 22 questions on food-related parenting practices situated within four higher-order theoretical domains, including Coercive Control (5 items), Indulgent (3 items), Structure (5 items), Autonomy support (9 items); the language for each individual measure is included in Table 2. Existing questionnaires including the Child Feeding Questionnaire (8) and the Food Parenting Inventory (23) were used where possible to adapt individual questions for use within an EMA protocol. For example, an item on the Child Feeding Questionnaire designed to measure parental pressure to eat reads, “I have to be especially careful to make sure my child eats enough”. This question was adapted for use within an EMA protocol to focus on a parent's specific behavior at the most recent meal or snack consumed by their child. The adapted question read, “Thinking of this meal or snack, did you have to encourage your child to eat more food than they wanted to?”. Parents responded yes/no for each item, following each eating occasion they shared with their child. Additional details on the development of this survey tool have been previously published (14).

**Table 2:** Individual items from the EMA-based real-time parent feeding practices survey.

**High level feeding domain**	**Specific feeding behavior**
	Thinking about this meal or snack, did you….(Response options yes/no)
**Structure**	
	Sit and eat with your child
	Choose where your child ate the meal or snack
	Choose what foods your child got to eat
	Closely monitor the type and amount of food eaten by your child
	Allow your child to choose what to eat, from several options you had already picked out
**Autonomy support**	
	Involve your child in deciding what foods they would eat
	Allow your child to take seconds if they asked for them
	Teach your child about why you wanted them to eat more of certain foods
	Teach your child about why you wanted them to eat less of certain foods
	Tell your child you wanted them to eat more of certain foods
	Encourage your child to try at least a small amount of all foods offered
	Negotiate with your child about how much food they needed to eat
	Negotiate with your child about what foods they needed to eat
	Tell your child you wanted them to eat less of certain foods
**Coercive control**	
	Have to encourage your child to eat more food than they wanted to
	Offer your child a treat or reward for eating more
	Have to make sure your child did not eat too much food
	Offer your child a treat or reward for trying a new food
	Trick or bribe your child into eating more than they wanted to
**Indulgent**	
	Choose to prepare separate food that knew your child would enjoy eating
	Allow your child to choose a separate meal or different food because they did not want to eat what was offered
	Give your child food in order to calm them down or help manage their behavior

Parents were asked to use their cell phone to respond to this survey following each of their child's eating occasions for which they were present for a data collection period of 10 days. Additional details included in the measures section of the manuscript.

*Parent stress* was assessed during signal-contingent EMA surveys by the following 10 items developed based on previous qualitative findings of momentary impacts on food parenting practices (6) and rated on a 5-point Likert scale (1-very slightly or not at all to 5-extremely): Felt like I didn't have enough time to get everything done that I needed to; Busy with a number of work or household activities; Busy with family or friend activities; Occupied by a special event; Down, sad or depressed; Stressed out; Worn out, tired or exhausted; Sick or under the weather; Constantly on-the-go; Disrupted by unexpected changes to my plan or routine. A total score was calculated as the sum of item scores; possible scores ranged from 10 to 50.

*Parent mood (i.e., Negative and Positive Affect)* was each assessed during signal-contingent EMA surveys by 20 items adapted from the short form of the Positive and Negative Affect Scale (PANAS) (24) for use within an EMA protocol (25) and rated on a 5-point Likert scale (1-very slightly or not at all to 5extremely). Negative Affect (10 items) included: Distressed, Upset, Guilty, Scared, Hostile, Irritable, Ashamed, Nervous, Jittery, Afraid. Positive Affect (10 items) included: Interested, Excited, Strong, Enthusiastic, Proud, Alert, Inspired, Determined, Attentive, Active. A total score for each scale was calculated as the sum of item scores; possible scores ranged from 10 to 50.

Child Mood (i.e., Negative and Positive Affect) was assessed during signal-contingent EMA surveys by asking parents to report on their child's mood using a total of 8 items adapted from the PANAS-C (26) for use within an EMA protocol and rated on a 5-point Likert scale (1-very slightly or not at all to 5-extremely). Positive Affect (4 items) were Happy, Joyful, Excited, and Energetic. Negative Affect (4 items) included Sad, Angry, Nervous, and Upset. A total score for each scale was calculated as the sum of item responses; possible scores ranged from 4 to 20.

*Child positive behaviors and negative behaviors* were assessed during signal-contingent EMA surveys by asking parents to report on their child's behavior using 7 items developed based on previous qualitative findings of momentary impacts on food parenting practices (6) and rated on a 5-point Likert scale (1-very slightly or not at all to 5 extremely). Positive Behaviors (2 items) were Well-behaved and Agreeable/Easy Going. Negative Behaviors (5 items) were Getting into trouble/Acting Out; Crabby; Fussy/Whiny; Out-of-control; Having a hard time sitting still/Hyper/Overly-energetic. A total score for each scale was calculated as the sum of item scores; possible scores for Positive Behaviors ranged from 2 to 10 and possible scores for Negative Behaviors ranged from 5 to 25.

*Demographics*. Child- (e.g., age, sex), parent- (e.g., age, sex, educational attainment), and family level (e.g., income, family structure) demographic characteristics were assessed via questions on the Kids EAT! baseline survey (14).

### Data analysis

To evaluate temporal ordering, data collected from EMA event prompts (i.e., participant initiated survey of food parenting practices used at specific eating occasions) were paired with data from EMA signal prompts (i.e., research-initiated survey of parent stress, parent and child mood, child behaviors) collected earlier in the same day for each participant. The event-signal pairs are constructed non-exclusively, meaning that every signal prompt is matched with all the later event prompts within the day, and vice versa. The mean within-pair time (i.e., time between signal prompt and reported eating occasion) for participants in our sample was 4.216 h (SD: 3.044 h); this time was shortest between signal prompts and breakfast (0.086 h) and longest between signal prompts and dinner (5.427 h). Event prompts that did not have a corresponding signal prompt from earlier within the same day were not included in the analysis. Similarly, signal prompts that did not have a corresponding event prompt later on within the same day were dropped. This process yielded one or more within-day signal-event pairs for each participant; participants without any pairs were excluded from the current analysis (*n* = 7) for a total analytical sample of 3,108 pairs of observations on 109 participants. Parents reported on a range of different types of eating occasions [mean eating occasions reported per day per participant = 1.961 (SD = 0.956)]; specifically, 30.3% of parents reported at least one breakfast meal (80 total signal-breakfast pairs), 82.6% reported at least one lunch meal (647 total signal-lunch pairs), 89.0% reported at least one dinner meal (1,172 total signal-dinner pairs), and 89.9% reported at least one snack (1,209 total signal-snack pairs).

To explore the relationship between the observed parent stress and mood, as well as child mood and behavior earlier in the day and the later use of food parenting practices, we fit linear mixed effect regression models for each of the 4 domains (as outcomes) and 7 signal predictors (as predictors of interest; parent stress, parent/child positive and negative mood, child positive and negative behavior). To minimize the model fitting and interpretational challenges of multicollinear explanatory variables (e.g., parent negative mood and child negative behavior), we fit separate regression models for all the combinations of predictors and outcomes, meaning that there are 28 mixed-effect regression models fitted, each with fixed effects as the parent education, income, one of the signal predictors, the time difference between the meal and mood, and random effects including the individual and time of day. Domain score outcomes were log-transformed after adding one to decrease heteroscedasticity and yield interpretation of effects on a percentage change scale; predictors were standardized so that a one-unit difference in the mood/stress predictor was a 1 standard deviation difference. Models were adjusted for highest parent education, household income, and time difference between the signal-event pair (continuous), and included random effect terms for participant and event time (12–6 AM, 6–12 PM, 12–6 PM, 6–12 AM). All models were fitted in R (4.0.2) using package “lme4” with *p*-values were calculated using package “lmerTest”.

## Results

### Parental momentary factors associated with food parenting practices

Greater parent positive mood earlier in the day was associated with the use of structured eating practices later in the day (details in Table 3). A one standard deviation difference in parent negative mood earlier in the day was associated with a decrease in use of feeding practices from within the structure domain later on that same day (−2.5%, *p* = 0.008), whereas greater parent positive mood earlier in the day was associated with an increase in use of structure later on that same day (+3.7%, *p* = 0.003). Parent mood (negative or positive) earlier in the day was not found to be significantly associated with use of coercive control, indulgent, or autonomy support feeding practices later that same day in this sample (all *p*-values >0.05).

**Table 3:** Adjusted temporal associations between parent- (mood, stress) and child- (mood, behavior) factors early in the day and food parenting practices (coercive, indulgent, structure, autonomy support) later that same day (*n* = 109 parent-child pairs; 3,108 eating occasions).

	**Coercive**		**Indulgent**		**Structure**		**Autonomy support**	
	**Regression coefficient**	***P*** **-value**	**Regression coefficient**	***P*** **-value**	**Regression coefficient**	***P*** **-value**	**Regression coefficient**	***P*** **-value**
**Parent factors**								
Positive parent mood	0.010	0.340	0.006	0.505	**0.037**	**0.003**	−0.004	0.807
Negative parent mood	−0.011	0.166	−0.008	0.264	–**0.025**	**0.008**	0.001	0.926
Parent stress	**0.032**	**<0.001**	**0.030**	**<0.001**	0.013	0.202	**0.056**	**<0.001**
**Child factors**								
Positive child mood	−0.009	0.299	0.006	0.448	**0.035**	**<0.001**	0.006	0.616
Negative child mood	0.00949	0.169	**0.013**	**0.025**	–**0.025**	**0.001**	0.009	0.329
Positive child behavior	−0.008	0.325	–**0.016**	**0.020**	0.008	0.399	–**0.032**	**0.002**
Negative child behavior	0.013	0.081	0.013	0.051	−0.014	0.123	**0.029**	**0.004**

Each number is the regression coefficient estimation of the fixed effect of the corresponding signal covariate with the corresponding domain outcome, adjusting for three fixed effect terms: highest parent education, household income, and time difference between the signal-event pair (continuous); and two random effect terms: participant and time of the day that the event happens (categorically by “12–6 AM”, “6–12 PM”, “12–6 PM”, “6–12 AM”).

Bold values indicate a *p*-value < 0.05. Numbers are rounded to even.

Greater parent stress earlier in the day was associated with an increase in the use of coercive control (+3.2%, *p* < 0.001), indulgent (+3.0%, *p* < 0.001) and autonomy support (5.6%, *p* < 0.001) feeding practices later on that same day.

### Child momentary factors associated with food parenting practices

As detailed in Table 3, child negative behavior earlier in the day was associated with greater use of autonomy support feeding practices. (+2.9%, *p* = 0.004). Greater child positive behavior earlier in the day was associated with a decrease in parent use of indulgent (−1.6%, *p* = 0.020) and autonomy-support (3.2%, *p* = 0.002) feeding practices later that same day. Child behavior (positive or negative) was not found to be significantly associated with use of coercive control or structured feeding practices later that same day (all *p*-values >0.05).

Child negative mood earlier in the day was associated with an increase in indulgent (+1.3%, *p* = 0.025) and a decrease in the use of structure (−2.5%, *p* = 0.001) feeding practices, whereas child positive mood earlier in the day was associated with an increase in the use of structured feeding practices (+3.5%, *p* < 0.001) later that same day. Child mood (positive or negative) was not found to be significantly associated with use of coercive control or autonomy support feeding practices later that same day within the current sample (all *p*-values >0.05).

## Discussion

The current study sought to understand momentary influences of parental stress, parent and child mood, and child behavior on parent's subsequent use of specific food parenting practices. Specifically, we hypothesized that higher stress, lower mood (parent or child), and worse child behavior earlier on in the day would be associated with increased use of less supportive parent feeding practices later on that same day. To our knowledge, the current study is the first one to examine momentary influences on the use of such a broad range of food parenting practices, including practices from across the four higher-order domains most commonly discussed in current conceptual models of food parenting practices (structure, autonomy support, coercive control, indulgence) (2). This study represents an important next step toward the future development of interventions to promote the use of supportive food parenting practices that are more responsive to free living environments including momentary change in context and circumstances. Overall, findings align with study hypotheses in that parent stress, parent and child low mood, and child negative behaviors early in the day were found to be associated with the use of less supportive food parenting practices later that same day; important nuances to these findings are discussed in detail below.

In alignment with study hypotheses, higher levels of parent stress early in the day was found to be associated with increased use of coercive control and indulgent feeding practices later that same day. These findings lend quantitative support to the findings stemming from our prior qualitative study in which parents who were interviewed described responding to stressful situations or circumstances by “downshifting” their mealtime interactions with their children away from aspirational efforts (high structure) toward more responsive feeding (coercive control, indulgence); specifically, findings from the current study provide evidence of temporal ordering of the momentary influence of stress on specific parent feeding practices (6). These findings also align, in part, with previous EMA studies by Berge and colleagues which found that parental stress experienced earlier in the day were associated with use of pressure-to-eat parenting practices at the evening meal; interestingly, Berge found no association between stress and food restriction, which is another aspect of coercive control food parenting (16, 17). The fact that the current study conceptualized individual coercive control behaviors (e.g., pressure-to-eat, restriction, threats/bribes) together under a single higher-order domain of coercive control, limits direct comparisons between studies. Findings from the current study also extend the prior work of Berge and colleagues, by examining the potential impact of stress on a wider range of stress on a broader range of parent feeding practices; future research should seek to replicate these findings, including examination of a similarly broad range of parent feeding practices. Further, future research should seek to specifically examine if the shift from more supportive—to less supportive—practices in the face of stressful circumstances, which was previously described by parents within qualitative research, can be observed using quantitative methods (6). Clinicians and public health practitioners may want to consider discussing with parents the impact that stress can have on their interactions with their child at subsequent mealtimes and work with parents to identify opportunities for stress reduction as well as the development of problem solving strategies to successfully navigate stressful situations as they arise.

Contrary to study hypotheses, greater levels of parental stress and negative behavior earlier in the day were all associated with greater parent use of autonomy support feeding practices later that same day. These findings suggest that when faced with greater challenges (i.e., stress, low poor, child negative behaviors) parents responded by increasing their engagement in feeding practices that supported their child's independence at mealtime, including behaviors such as involving their child in choosing what they wanted to eat, teaching them about the benefits and drawbacks of certain foods, and engaging in encouragement or negotiation regarding the types and amounts of foods eaten by their child at meals. The current study does not shed light on why parents chose to increase engagement in autonomy support practices and these findings feel particularly challenging to interpret given that it feels somewhat counterintuitive to see increases in autonomy support and coercive control in response to higher levels of parent-reported stress. That said, it might be that parents in this sample responded to high stress or otherwise challenging situations by moving away from maintaining some of the more covert- or structure-based food parenting practices and toward more overt food parenting practices, including practices from within both the autonomy support and coercive control domains. A parent experiencing high stress or whose child is experiencing low mood or behavior-related challenges might feel less equipped to maintain their usual level of structure a mealtimes, and respond instead by engaging in more direct goal-oriented interactions with their child around food at mealtimes. This direct interaction could look like autonomy support behaviors, coercive control behaviors, or both depending on the individual family circumstances and skills sets. For example, if early morning stress challenged a parent's ability to meal plan, they might lean more on including their child in helping them make these decisions in an effort to complete the task and to be inclusive of the child in a way that could promote more positive interactions at the future meal; alternatively, another parent experiencing a stressful day might respond by engaging in pressure-to-eat with the goal of rushing their child through the meal to “get it over with”.

Future studies should aim to replicate the findings that greater levels of parental stress and negative behavior earlier in the day were all associated with greater parent use of autonomy support feeding practices and seek to deepen our understanding of the connection between challenging circumstances and parents increased use of autonomy support practices; this deepened understanding of the mechanisms at play will be key in future intervention development. Public health practitioners should seek to explore ways they can help support families in maintaining structure in the face of challenging circumstances, as well as encourage families to choose autonomy support practices over coercive control practices when possible. It is also important to note that while autonomy support behaviors has been identified as supportive of the development of healthful eating patterns and dietary intake in young children (2), the specific feeding practices that make up this higher-order domain have been studied far less that other specific feeding practices (e.g., pressure-to-eat, restriction, availability, accessibility) (2–5). Further, it is possible that items developed to measure autonomy support within the Real-time Parent Feeding Practices survey tool (14) are indeed measuring a parenting practice that better aligns with a different higher-order domain (e.g., coercive control). For example, parents might interpret what “negotiate” means differently than current theoretical models and researchers intend them to. It is crucial that future research continue to understand which specific aspects of autonomy support associated with healthful dietary intake overtime in young children to allow for the development of interventions tailored to promote food parenting practices most supportive of positive child outcomes overtime.

There are both strengths and limitations to this study. First, this study adds significantly to the emerging literature aimed at broadening our conceptualization of food parenting practices, by being the first, to our knowledge, to examine momentary influences on the use of such a broad range of food parenting practices, including practices from across the four higher-order domains most commonly discussed in current conceptual models of food parenting practices (structure, autonomy support, coercive control, indulgence) (2). Additionally, this study was able to assess the impact of a range of momentary predictors, including both parent (stress and mood) and child (mood and behavior) factors. Further, while the overall sample size of this study was small (*n* = 109), the ability to use data from each single-event reported *via* EMA resulted in a total of 3,108 signal-event-pairs for analysis, which is a strength of this data collection approach. EMA data collection, including measures of parental stress, parent and child mood, child behaviors and food parenting practices are reliant on parent self-report which may introduce some social desirability bias to responses. However, repetitive, real-time reporting of feeding practices represents a move away from gathering parent report of aspirational perceptions and enables us to capture more variation in behaviors by not asking parents to reduce their actual practices down to a single average response (6, 14). It is possible that repetitive data collection and reporting on one's own behavior might act as a mini-intervention, leading parents to change their behavior over the course of the data collection time period.

## Conclusion

The current study sought to understand how parent stress, parent and child mood and child eating behaviors early in the day are temporally associated with parent's use of specific food parenting practices later that same day. This study represents an extension of recent research which has highlighted that food parenting practices are not static behaviors, rather they are context specific and responsive to momentary factors within the home and family environment. Findings from the current study support and extend prior research support prior finding indicating that parent and child mood, stress and child behavior earlier in the day are associated with parent's use of specific food parenting practices later in that same day. Currently, clinical and public health recommendations made to parents largely overlook the impact of momentary contextual influences on food parenting practices. By identifying circumstances in which parents are most likely to struggle to use supportive feeding practices, findings from the current study can inform the development of just-in-time adaptive interventions aimed at supporting parents' use of food parenting practices that are supportive of healthful child dietary intake and eating behaviors in a way that is responsive to shifting momentary factors.

## Data availability statement

The raw data supporting the conclusions of this article will be made available by the authors, without undue reservation.

## Ethics statement

The studies involving human participants were reviewed and approved by University of Minnesota Twin Cities. Written informed consent to participate in this study was provided by the participants' legal guardian/next of kin.

## Author contributions

KL is the principal investigator for the Kids EAT! study, conceptualized the paper, assisted with data interpretation, and worked collaboratively with all co-authors to write the paper. ZJ conducted the data analysis. JW oversaw the data analysis conducted by ZI. JB and JF provided mentorship throughout the conceptualization of the Kids EAT! research study and critically reviewed all drafts of the paper. DN-S provided mentorship throughout the conceptualization of the Kids EAT! research study and is the principal investigator for the EAT 2010–2018 study, the larger study from which the study participants were recruited. All authors assisted with conceptualization of the paper, critically reviewed the full paper, gave final approval this version to be published, and agreed to be accountable for all aspects of the work regarding the accuracy or integrity of any part of the work.
